# Mission vs. Margin: The Effects of Catholic Health System Ownership on Hospital Operations

**DOI:** 10.1177/10775587251355541

**Published:** 2025-07-24

**Authors:** Alex Schulte, Becky Staiger, Hector P. Rodriguez, Amanda L. Brewster

**Affiliations:** 1 University of California, Berkeley, USA

**Keywords:** health systems, hospital operations, ownership, acquisition, Catholic

## Abstract

The number of Catholic hospitals grew by 28% between 2001 and 2020, and today almost one-fifth of U.S. nonprofit hospitals are Catholic. Catholic systems face conflicting institutional pressures to provide mission-oriented services while remaining financially competitive. Using 2009–2022 data from the American Hospital Association (*n* = 33,552 hospital-years), we applied difference-in-differences methods to compare changes in hospital operations after acquisition by Catholic and non-Catholic systems relative to the control group of never-acquired hospitals. Catholic-acquired hospitals were more likely to offer some mission-oriented services, including chaplaincy and charity care (average treatment effect on the treated, ATT, 10.41 percentage-point [pp] and 3.97 pp, respectively), while non-Catholic-acquired hospitals were less likely to operate an obstetrics unit (ATT −3.45 pp) after acquisition relative to the control group. Hospitals experienced similar cost-cutting measures after acquisition relative to the control group, including reduced operating expenses and employment, regardless of system ownership type. Our findings provide rigorous empirical evidence to inform ongoing policy debates regarding the expansion of Catholic health care.

## Introduction

Perhaps best summarized by the phrase “no margin, no mission,” Catholic health systems face conflicting pressures to maintain their mission-oriented identity while staying financially viable in the competitive, profit-driven American health care landscape. Trends in consolidation and acquisition have accelerated in recent decades as health systems—both Catholic and non-Catholic—seek to expand their market power and financial strength (Andreyeva et al., 2024; Burns et al., 2015; Homan & White, 2021; Lee & Alexander, 1999). The number of Catholic-owned or affiliated hospitals grew by 28% between 2001 and 2020, and today almost one-fifth of U.S. nonprofit hospitals are Catholic (Solomon et al., 2020). Catholic hospitals are also larger, with more beds on average, than their non-Catholic counterparts (Cartwright et al., 2023; Solomon et al., 2020). Especially in rural areas, patients may have to travel long distances, if that is feasible, to access non-Catholic hospitals (Kramer et al., 2021; Solomon et al., 2020).

Catholic hospitals adhere to the Ethical and Religious Directives (ERDs), a list of 77 specific directives that govern hospital operations and clinical care (United States Conference of Catholic Bishops, 2018). The ERDs emphasize Catholic health care’s commitment to serving poor and vulnerable populations, including the uninsured, racial/ethnic minorities, immigrants, Medicaid beneficiaries, and rural populations (Doderer, 2011; Haddad, 2023; Hochman, 2017). In addition, Catholic hospitals are expected to provide pro-natalist obstetrics care to support “the sanctity of human life from its very beginning.” However, the ERDs also explicitly forbid Catholic hospitals to provide services such as abortion, contraception, and sterilization that “render procreation impossible” (United States Conference of Catholic Bishops, 2018).

Previous research has found Catholic hospitals were more likely to provide charity care and community benefit services compared to non-religious nonprofit hospitals (Ferdinand et al., 2014; Zare & Gabow, 2023), and more likely to offer stigmatized services (e.g., HIV/AIDS, psychiatric care) compared to public and for-profit hospitals (White et al., 2006). Some studies also suggest that Catholic hospitals may be more likely to provide obstetrics care and operate a neonatal intensive care unit (Garrido et al., 2012; Weisman et al., 1999). Given the increasing trend of obstetric unit closures and consolidation, especially in rural areas, this suggestive evidence warrants further investigation (Fischer et al., 2024; Henke et al., 2021; Kozhimannil et al., 2020).

However, research has also found that organizational structure, management, and patient experience within modern Catholic hospitals have become increasingly similar to their non-Catholic counterparts (Homan & White, 2021; Kutney-Lee et al., 2014; O’Loughlin, 2024). This convergence aligns with broader trends in the health care industry, where acquisitions often lead to significant operational changes at acquired facilities (Burns et al., 2015; Furukawa et al., 2020; Lee & Alexander, 1999). For example, Andreyeva et al. found that operating expenses declined after previously independent hospitals in New York state joined a health system, and these declines were primarily driven by a decrease in the number of employees concentrated in support functions. However, patient volume remained unchanged, suggesting that large systems were able to increase efficiency—reduce inputs while maintaining the same level of output (Andreyeva et al., 2024).

### New Contribution

A recent literature review found that, despite the significant role of Catholic providers within the U.S. health care system, there are relatively few studies on this topic (Thorne et al., 2019). Previous research on Catholic health care is mainly descriptive in nature and limited by the potential for selection bias and unobserved confounding. In this study, we advance the literature by applying rigorous quasi-experimental methods to analyze the impact of competing institutional logics within Catholic health systems. By comparing changes in hospital operations following acquisitions by different ownership types, we empirically assess how Catholic health systems navigate competing mission-driven and financial tensions differently than their non-Catholic counterparts. Primary hospital operation outcomes are mission-oriented services (e.g., chaplaincy, charity care) and obstetrics care—services that are typically not profitable, but Catholic systems may be more likely to provide given their religious doctrine (Haddad, 2023; Panicola & Hamel, 2015). Secondary hospital operation outcomes are measures of utilization, operating expenses, and employment, which may change with the financial pressures associated with health system acquisitions (Andreyeva et al., 2024; Burns et al., 2015).

Given the expansion of Catholic health care, our findings will inform current state policy debates on institutional conscience rights and government oversight in health care acquisitions. For example, several states are considering bills with additional protections or requirements related to the “institutional conscience” rights of Catholic hospitals in the wake of the *Dobbs vs. Jackson Women’s Health Organization* Supreme Court decision (Sawicki, 2024). In addition, we make novel contributions in organizational theory by using the case of Catholic system acquisitions to illustrate how mission-driven organizations balance competing institutional logics, which can be defined as the organizing principles associated with different aspects of society that guide organizational behavior (Friedland & Alford, 1991; Singer et al., 2020).

### Conceptual Model

In recent decades, there has been increasing interest in extending neo-institutional theory to study the multiple (and oftentimes, competing) institutional logics that drive organizational behavior, especially during times of organization change (Franz et al., 2021; Goodrick & Reay, 2016; Thornton & Ocasio, 1999). Catholic acquisitions present an interesting case to study the impact of applying multiple institutional logics—for example, religious and financial logics— within newly acquired hospitals. Not only do Catholic hospitals adhere to the ERDs, an explicit set of rules and values governing their operations, they also have organizational resources and incentives to follow these directives. When a hospital is acquired by a Catholic health system, hospital leadership and staff typically go through a series of trainings to ensure alignment with the system’s Catholic values, brand identity, and operating model. Catholic systems also have a “Director of Mission Integration” who uses established tools, such as the Catholic Ministry Identity Assessment, to oversee the consistent implementation of the ERDs in practice (Panicola & Hamel, 2015; Santos, 2023; Smith, 2018). Moreover, Catholic health care systems often rely on support and funding from local communities and the Catholic Church (Catholic News Agency Staff, 2021; Gagliarducci, 2021; O’Loughlin, 2024). By consistently applying the shared rules and values outlined in the ERDs, Catholic hospitals signal to stakeholders—patients, the Catholic Church, and the broader community—that the organization remains faithful to its identity and is “deserving” of resources. This context informs our first hypothesis:

**Hypothesis 1:** Hospitals are more likely to preserve or expand mission-oriented services and obstetrics care after joining Catholic systems compared to non-Catholic systems.

At the same time, the U.S. health care landscape has become increasingly competitive and profit-driven in recent years, contributing to accelerated merger/acquisition trends to expand providers’ market power and financial stability (Andreyeva et al., 2024; Burns et al., 2015; Lee & Alexander, 1999). Policymakers and regulators are increasingly scrutinizing hospital acquisitions due to concerns over profit-driven incentives and potential reductions in access to care, especially in rural and underserved areas (Furukawa et al., 2020; Fuse Brown & Gudiksen, n.d.). Today, four of the 10 largest U.S. health care systems are Catholic, including the single largest nonprofit system, CommonSpirit Health (Solomon et al., 2020). To remain competitive and financially viable, health systems—both Catholic and non-Catholic—must operate efficiently and look for opportunities to cut costs, especially within newly acquired hospitals (Andreyeva et al., 2024; Burns et al., 2015). This context informs our second hypothesis:

**Hypothesis 2:** Post-acquisition, hospitals implement cut-cutting measures, including lower operating expenses and decreased employment, while maintaining or increasing utilization. These effects are similar after both Catholic and non-Catholic system acquisitions.

## Methods

### Data and Study Sample

We integrated five data sources to construct a 14-year (2009–2022) unbalanced panel dataset of U.S. hospitals. Our primary data source was the American Hospital Association (AHA) survey, a comprehensive survey of U.S. hospitals is completed annually by hospital administrators and contains information on hospital location, system membership, size, entity type (government, for-profit, or nonprofit), utilization, expenses, employment, and service lines. Consistent data collection and survey processes are used to facilitate accurate time-series analyses (AHA Data & Insights, 2025). The inclusion of a unique, stable hospital identifier allows hospitals to be linked over time and analyzed longitudinally. 78% of hospitals in our sample had data for all 14 years (2009–2022). Hospital closures and openings mean that some hospitals were added or removed from the dataset each year. The survey typically achieves high response rates (above 75%). If a hospital was open, but did not respond to the survey in a given year, AHA imputes missing values using a structured estimation process based on historical data and peer hospitals, creating a high degree of continuity across years (AHA Data & Insights, 2025; CDC National Center for Health Statistics, 2024).

We use several secondary data sources to check and confirm data on the acquisition of hospitals by health systems, including the LevinPro HC database that tracks health care mergers and acquisitions, the Centers for Medicare and Medicaid Hospital Change of Ownership dataset, and Health Care Pricing Project data on hospital acquisitions (Cooper et al., 2022). We also used the Compendium of U.S. Health Systems maintained by the Agency for Health care Research and Quality to identify religiously-affiliated health systems. We then manually validated if each religiously-affiliated system was Catholic using hospital and health system websites, press releases, and local diocesan lists of affiliated organizations.

The initial dataset included 86,571 total hospital-years. To construct the analytic sample, we excluded hospitals that were part of a health system throughout the study period (*n* = 49,854). It is necessary to exclude these “already-treated” hospitals to maintain a clean control group and ensure the validity of difference-in-differences (DID) estimates (Callaway & Sant’Anna, 2021). In addition, we excluded hospitals that switched in or out of a system more than once (*n* = 1,033), were part of both Catholic and non-Catholic systems over the study period (*n* = 181), and had less than 2 years of data before or after joining a system (*n* = 1,951). The final analytic sample included 2,722 unique hospitals and 33,552 hospital-years from 2009 to 2022. During the study period, there was a total of 707 first-time system acquisitions of previously independent hospitals, 76 (11%) by Catholic systems, and 631 (89%) by non-Catholic systems.

### Measures

#### Treatment Variable

Treatment is defined as the first year a previously independent hospital was acquired by a health system (a central organization that owns and/or manages health provider facilities or health-related subsidiaries). We only analyzed first-time acquisitions to ensure that any observed changes could be clearly attributed to the impact of joining a particular health system, without potential for contaminating effects from previous acquisitions. To explore if treatment effects differed depending on whether the acquiring health system was Catholic vs. non-Catholic, we further identified two treated sub-groups: “Catholic-acquired” (hospitals whose first acquisition was by a Catholic system) and “non-Catholic-acquired” (hospitals whose first acquisition was by a non-Catholic system). For all analyses, the control group was consistently defined as hospitals that were independent throughout the study period (“never-acquired” hospitals).

#### Primary Outcomes

Our primary hospital operation outcomes were measures of mission-oriented services and obstetrics care. Mission-oriented services were dichotomous variables indicating the provision of the following four services at the end of the fiscal year: (a) chaplaincy, defined as religious ministering and pastoral counseling services for hospital patients, their families, and staff; (b) charity care, defined as health care services provided free of cost or on a sliding scale for uninsured and underinsured persons, including free clinics subsidized by the sponsoring health care organization; (c) community outreach, defined as a hospital program that systematically interacts with the community to identify those in need of services, alerting persons and their families to the availability of services, locating needed services, and enabling persons to enter the service delivery system; and (c) linguistic/translation, defined as services to make health care more accessible to non-English speaking patients.

Considering Catholic hospitals’ stated commitment to ensuring access to obstetrics care, alongside the recent trend of obstetric unit closures, we also examined changes in obstetrics care following acquisitions by Catholic and non-Catholic systems. An obstetrics unit was defined as a hospital unit that provided care, examination, treatment, and other services to women during pregnancy, labor, and postpartum. Other obstetrics care outcomes included obstetric bed count and proportion of obstetric beds to total hospital beds.

#### Secondary Outcomes

Secondary hospital operation outcomes included measures of utilization, operating expenses, and employment. Bed count was defined as number of beds set up and staffed at the end of the fiscal year. Bed count could be both a predictor of acquisition and/or change significantly post-acquisition. Therefore, we normalized the subsequent outcomes by bed count to eliminate heterogeneity purely due to differences in hospital size. Utilization measures per bed included: total inpatient admissions and number of inpatient days with Medicaid as the primary payer and, separately, Medicare. We measured the following three types of operating expenses per bed: (a) total expenses, defined as all operating expenses during the fiscal year, including payroll, employee benefits, and all other expenses; (b) payroll expenses, which included all salaries and wages; and (c) employee benefit expenses, which included social security, group insurance, retirement benefits, workman’s compensation, and unemployment insurance. Employment was measured as the number of full-time equivalents (FTEs) per bed on payroll, which accounted for both full-time and part-time employees. Doctors of Medicine (MDs) included physicians and dentists. Nurses included registered, licensed practical, and vocational nurses. Support staff included assistive nursing personnel, technicians (radiology, laboratory, pharmacy), respiratory therapists, and all other employees.

#### Covariates

Our analyses controlled for Medicaid payer mix (defined as proportion of total inpatient days with Medicaid as the primary payer) for both conceptual and analytical reasons. We have conceptualized Medicaid payer mix as a confounder, given it may impact both treatment assignment (hospital acquisition) and study outcomes (mission-oriented services, obstetrics care, etc.). Our statistical tests confirmed this variable was associated with lower odds of hospital acquisition (see Supplemental Appendix A) and also changed differentially over time between the treatment and controls groups in the pre-period (e.g., non-parallel pretrends, see Supplemental Appendix B). Therefore, we have included Medicaid payer mix as a covariate to more precisely isolate acquisition treatment effects. We did not control for Medicaid payer mix when analyzing Medicaid days per bed or Medicare days per bed due to collinearity.

### Analysis

Our main analytical strategy aimed to estimate differences in hospital response to acquisitions by Catholic and non-Catholic systems. We also used a targeting regression to examine which types of hospitals were targeted for acquisition by Catholic and non-Catholic systems (additional details and results of the targeting regression are presented in Supplemental Appendix A). For our main analyses, we used a staggered DID specification to compare changes in trends at independent hospitals that were acquired by Catholic and non-Catholic systems (treated groups) vs. trends for independent hospitals that were never acquired (control group). Using a DID approach in which the “event” is the year of hospital acquisition, we compared changes in hospital operation measures between the 8 years before (“pre-period”) and 8 years after (“post-period”) acquisition, relative to trends in these measures among never-acquired hospitals over the same time period. Our model is as follows:

(1) $$\begin{array}{l} \gamma_{\mathrm{ht}}=\beta_{1}{\left(\mathrm{post}*\mathrm{Catholic}-\mathrm{acquired}\right)}_{\mathrm{ht}}+\\ \beta_{2}{\left(\mathrm{post}*\mathrm{non}-\mathrm{Catholic}-\mathrm{acquired}\right)}_{\mathrm{ht}}+\\ \gamma X_{\mathrm{ht}}+\theta_{h}+\theta_{t}+\in_{ht} \end{array}$$

ϒ_ht_ denotes the outcome of interest for hospital *h* in year *t*. β_1_ and β_2_ capture the average treatment effect on the treated (ATT) after joining Catholic and non-Catholic systems, respectively. *X*_ht_ denotes the time-varying hospital covariate, Medicaid payer mix. The model includes hospital fixed effects (θ_h_) to adjust for time-invariant hospital characteristics that may be correlated with the outcome (e.g., geographic location), and year fixed effects (θ_t_) to adjust for national shocks (e.g., economic conditions, COVID-19 pandemic). Standard errors were clustered at the hospital level. We “winsorized” each continuous outcome, meaning outlier observations below the first percentile and above the 99th percentile were replaced with the 1st and 99th percentile values, respectively, given these values were likely not feasible.

Our preferred specification utilized the Borusyak et al. imputation-based DID approach, which is particularly well-suited to our empirical setting given its efficient handling of both time- and group-based heterogeneous treatment effects (Borusyak et al., 2024). Regarding time-based heterogeneity, hospital acquisitions occurred at different points over the study period (are “staggered”), so dynamic treatment effects may vary based on calendar year and/or relative time post-acquisition. Recent literature has highlighted limitations of using traditional two-way fixed effects estimators with staggered treatment timing (Johansson et al., 2024; Wang et al., 2024; Wing et al., 2024). By constructing imputed untreated outcomes for each treated unit (using pre-treatment data and the never-treated control group), the Borusyak et al. method accommodates complex patterns in treatment timing. This approach mitigates concerns with staggered timing and enhances both the precision and efficiency of our estimates (Borusyak et al., 2024). In addition, the Borusyak et al. approach allows us to assess the statistical significance of group-based heterogeneous effects (here, Catholic vs. non-Catholic system acquisitions), a capability not directly available with other estimation procedures that do not use an imputation approach. Last, the Borusyak et al. method requires a stronger parallel trends assumption compared to alternative approaches, which enhances the credibility of causal effects if such assumptions are met.

In our model, the identifying variation was based on the staggered timing of hospital acquisitions and the comparison of acquired and never-acquired hospitals in each relevant time period. To interpret β_1_ and β_2_ as the causal effect of system acquisition on outcome ϒ_ht_, we must assume that the outcome trends of the acquired hospitals would have been parallel to the outcome trends of the never-acquired hospitals in the absence of treatment. We assessed pre-treatment trends both formally (using joint pretrends tests) and graphically (using event study plots) and confirm the parallel trends assumption was met for all study outcomes. In Supplemental Appendix Table B1, we present *p*-values from formal statistical tests of jointly significant pretrends for each outcome. Joint *p*-values of >.05 support the parallel pretrends assumption, as we are unable to reject the null hypothesis of no differences in trends between treatment and control groups prior to acquisition. Given the staggered nature of the treatment timing, we also present event study plots to observe how treatment effects vary in the years before and after joining a health system.

We ran several robustness checks to test the sensitivity of our findings to study design decisions. Specifically, we ran the following analyses: (a) standard two-way fixed effects DID estimator rather than the imputation-based approach, (b) limiting the pre- and post-periods to 6 years instead of 8, (c) excluding Medicaid payer mix as a covariate, and (d) including hospital entity type (government, nonprofit, for-profit) as a covariate. We chose not to include entity type as a covariate in our main DID model because this variable is most likely to change after hospital acquisition, so including it as a covariate risks biasing the acquisition treatment effect (Horwitz, 2005; Wang et al., 2024). All analyses were performed in Stata 18. *P*-values < .05 were considered statistically significant. The University of California, Berkeley institutional review board determined the research protocol to be exempt (#2024-08-17681).

## Results

### Baseline Summary Statistics

At the start of the study period, a higher proportion of hospitals later acquired by non-Catholic systems provided mission-oriented services and obstetrics care compared to hospitals later acquired by Catholic systems and never-acquired hospitals (Supplemental Appendix Table A2). Bed count and operating expenses were also highest at the start of the study period at hospitals later acquired by non-Catholic systems, but total FTEs per bed were highest at hospitals later acquired by Catholic systems. By incorporating hospital fixed effects in Equation 1, our DID model relies on “within-hospital” comparisons, effectively controlling for any time-invariant hospital characteristics that drive these baseline differences and may be correlated with our outcomes of interest.

### Primary Outcomes

**Figure 1.**
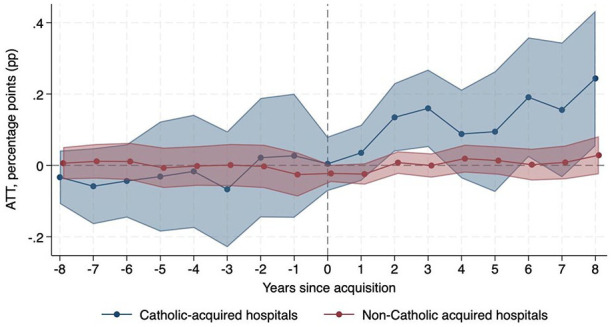
Change in Probability of Providing Chaplaincy Care Relative to Never-Acquired Hospitals.

**Figure 2.**
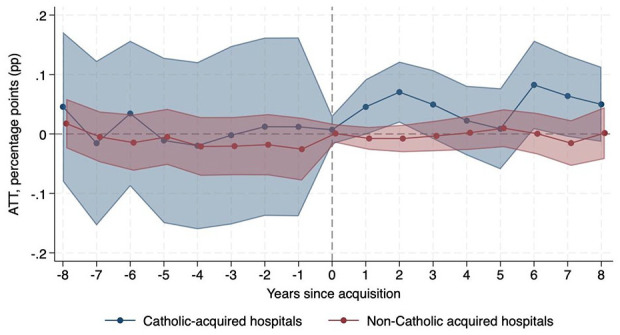
Change in Probability of Providing Charity Care Relative to Never-Acquired Hospitals.

Table 1 presents results from Equation 1, the average treatment effects of Catholic (col. 1) and non-Catholic (col. 2) acquisitions for our primary outcomes. We also present the difference in effect magnitudes between Catholic vs. non-Catholic acquisitions (col. 3). Following acquisition, Catholic-acquired hospitals experienced a relative increase in mission-oriented services compared to hospitals that were never acquired, including a 10.41 percentage-point (pp) (95% CI 1.36, 19.46) relative increase in the provision of chaplaincy care and a 3.97 pp (95% CI 0.40, 7.54) relative increase in charity care. Non-Catholic-acquired hospitals did not experience a change in chaplaincy or charity care compared to never-acquired hospitals. The difference in treatment effect magnitudes between Catholic-acquired and non-Catholic-acquired hospitals for chaplaincy and charity care was statistically significant (Table 1, col. 3). Figures 1 and 2 show the dynamic effects for trends in chaplaincy and charity care, respectively, over time, which align with these DID results. These event study plots, along with Supplemental Appendix Table B1, confirm the parallel trends assumption was met. We do not observe any statistically significant changes in community outreach or linguistic/translation services after Catholic or non-Catholic acquisitions.

**Table 1.** Treatment Effects After Catholic and Non-Catholic Acquisitions for Primary Outcomes.

Variables	(1)	(2)	(3)
	Catholic-acquired	Non-Catholic-acquired	Difference between Catholic-acquired and non-Catholic-acquired
**Mission-oriented services**			
**Chaplaincy**	0.10*	0.0008	0.10*
	(0.05)	(0.01)	(0.05)
	[0.01, 0.19]	[−0.03, 0.03]	[0.01, 0.20]
**Charity care**	0.04*	−0.002	0.04*
	(0.02)	(0.01)	(0.02)
	[0.004, 0.08]	[−0.02, 0.02]	[0.002, 0.08]
**Community outreach**	−0.005	−0.02	0.02
	(0.04)	(0.02)	(0.04)
	[−0.09, 0.08]	[−0.05, 0.006]	[−0.07, 0.11]
**Linguistic/translation services**	0.02	0.01	0.01
	(0.04)	(0.02)	(0.04)
	[−0.06, 0.11]	[−0.02, 0.04]	[−0.08, 0.10]
**Obstetrics**			
**Obstetrics unit**	−0.04	−0.03*	−0.01
	(0.03)	(0.01)	(0.03)
	[−0.09, 0.01]	[−0.06, −0.01]	[−0.06, 0.05]
**Obstetric bed count**	−0.79	−0.87***	0.07
	(0.42)	(0.22)	(0.47)
	[−1.62, 0.04]	[−1.30, −0.44]	[−0.84, 0.99]
**Proportion obstetric beds/total beds**	−0.005	−0.001	−0.004
	(0.004)	(0.002)	(0.005)
	[−0.01, 0.004]	[−0.005, 0.002]	[−0.01, 0.006]

*Note*. Table 1 presents results from Equation 1, which estimates the average treatment effects of Catholic (col. 1) and non-Catholic (col. 2) acquisitions, for our primary outcomes. Column (3) uses an imputation approach to estimate the difference between Catholic-acquired (col. 1) and non-Catholic-acquired (col. 2) treatment effects. The model includes hospital and year fixed effects and controls for Medicaid payer mix. All outcomes presented meet the parallel trends assumption. Standard errors are clustered at the hospital level and presented in parentheses. 95% confidence intervals are presented in brackets.

* *p* < .05. **p < .01. ***p < .001.

**Figure 3.**
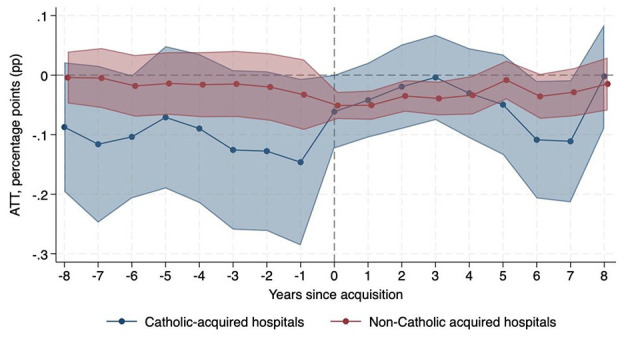
Change in Probability of Operating an Obstetrics Unit Relative to Never-Acquired Hospitals.

**Figure 4:**
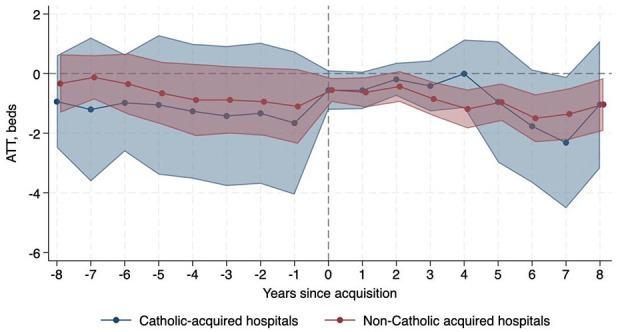
Change in Number of Obstetric Beds Relative to Never-Acquired Hospitals.

Regarding obstetrics care, non-Catholic-acquired hospitals were relatively 3.45 pp (95% CI −5.70, −1.21) less likely to operate an obstetrics unit after acquisition than never-acquired hospitals, while there was no significant change in probability among Catholic-acquired hospitals. In addition, obstetric bed count decreased after non-Catholic acquisitions (ATT −0.87, 95% CI −1.30, −0.44) compared to never-acquired hospitals, but there was no significant change after Catholic acquisitions. The proportion of obstetric beds to total beds did not change at Catholic-acquired or non-Catholic-acquired hospitals relative to never-acquired hospitals. Figures 3 and 4 show the decrease in probability of operating an obstetrics unit and obstetric bed count, respectively, after non-Catholic acquisitions.

### Secondary Outcomes

Table 2 presents the average treatment effects of Catholic and non-Catholic acquisitions for our secondary outcomes. Total bed count decreased by 12.41 (95% CI −18.44, −6.37) and 4.31 (95% CI −7.95, −0.68) for Catholic-acquired and non-Catholic-acquired hospitals, respectively, relative to never-acquired hospitals; these treatment effect magnitudes differed by −8.09 (95% CI −14.86, −1.32). Supplemental Appendix Figure C1 is consistent with these ATT estimates and show larger decreases over time, indicating more substantial long-run effects. The significant treatment effects for bed count reinforce the importance of adjusting for bed count in the subsequent outcomes.

**Table 2.** Treatment Effects After Catholic and Non-Catholic Acquisitions for Secondary Outcomes.

Variables	(1)	(2)	(3)
	Catholic-acquired	Non-Catholic-acquired	Difference between Catholic-acquired and non-Catholic-acquired
**Utilization**			
**Bed count**	−12.41***	−4.31*	−8.09*
	(3.08)	(1.85)	(3.45)
	[−18.44, −6.37]	[−7,95, −0.68]	[−14.86, −1.32]
**Admissions per bed**	−1.35	1.39***	−2.73*
	(1.02)	(0.44)	(1.09)
	[−3.35, 0.66]	[0.52, 2.25]	[−4.88, −0.59]
**Medicaid days per bed**	4.64	4.14*	0.50
	(2.49)	(1.16)	(2.66)
	[−0.24, 9.52]	[1.86, 6.42]	[−4.71, 5.70]
**Medicare days per bed**	−6.28*	4.10***	−10.37***
	(2.90)	(1.32)	(3.10)
	[−11.96, −0.60]	[1.50, 6.70]	[−16.45, −4.30]
**Operating expenses**			
**Total expenses per bed**	30,818.84	25,767.52	5051.32
	(39,038.36)	(16,483.95)	(40,805.85)
	[−45,694.93, 107,332.60]	[−6,540.431, 58,075.47]	[−74,926.68, 85,029.32]
**Payroll expenses per bed**	−6,057.64	−5,285.82	−771.81
	(15,270.27)	(6,281.09)	(15,885.04)
	[−35,986.81, 23,871.54]	[−17,596.54, 7,024.89]	[−31,905.92, 30,362.29]
**Employee benefit expenses per bed**	−3,496.23	−4,890.66*	1,394.43
	(4,298.94)	(1,706.54)	(4,452.17)
	[−11,922.00, 4,929.55	[−8,235.41, −1,545.91]	[−7,331.67, 10,120.53]
**Employment**			
**Total FTEs per bed**	−0.39	−0.26***	−0.13
	(0.27)	(0.09)	(0.27)
	[−0.91, 0.13]	[−0.43, −0.08]	[−0.67, 0.40]
**MD FTEs per bed**	−0.03*	−0.02***	−0.02
	(0.01)	(0.006)	(0.01)
	[−0.06, −0.01]	[−0.03, −0.006]	[−0.04, 0.009]
**Nurse FTEs per bed**	0.01	0.02	−0.01
	(0.02)	(0.02)	(0.07)
	[−0.03, 0.07]	[−0.03, 0.07]	[−0.14, 0.013]
**Support staff FTEs per bed**	−0.36	−0.24***	−0.12
	(0.20)	(0.07)	(0.21)
	[−0.77, 0.03]	[−0.37, −0.11]	[−0.53, 0.28]

*Note*. Table 2 presents results from Equation 1, which estimates the average treatment effects of Catholic (col. 1) and non-Catholic (col. 2) acquisitions, for our secondary outcomes. Column (3) uses an imputation approach to estimate the difference between Catholic-acquired (col. 1) and non-Catholic-acquired (col. 2) treatment effects. The model includes hospital and year fixed effects and controls for Medicaid payer mix. All outcomes presented meet the parallel trends assumption. MD, Doctor of Medicine (includes physicians and dentists); FTE, full-time equivalents. Standard errors are clustered at the hospital level and presented in parentheses. 95% confidence intervals are presented in brackets.

* *p* < .05. **p < .01. ***p < .001.

Efficiency of bed use, as measured by admissions per bed, increased by 1.39 after non-Catholic acquisitions (95% CI 0.52, 2.25) relative to never-acquired hospitals. Admissions per bed decreased after Catholic acquisitions, although this result is not statistically significant. The difference in treatment effect magnitudes for admissions per bed in Catholic-acquired vs. non-Catholic-acquired hospitals was −2.73 (95% CI −4.88, −0.59); Supplemental Appendix Figure C2 shows these divergent trends after acquisition. Medicaid days per bed increased by over 4 days after both Catholic and non-Catholic acquisitions relative to never-acquired hospitals, although only the change at non-Catholic-acquired hospitals was significant (ATT 4.14; 95% CI 1.86, 6.42). Medicare days per bed increased by 4.10 (95% CI 1.50, 6.70) after non-Catholic acquisitions but decreased by 6.28 (95% CI −11.96, −0.60) after Catholic acquisitions relative to never-acquired hospitals. The difference in Medicare days per bed effect magnitudes between Catholic-acquired vs. non-Catholic-acquired hospitals was −10.37 (95% CI −16.45, −4.30). Supplemental Appendix Figures C3 and C4, respectively, are consistent with these Medicaid and Medicare days per bed DID results and show increasing effects over time.

In contrast to these differing effects between Catholic-acquired vs. non-Catholic-acquired hospitals, changes in operating expenses were similar (although generally not statistically significant) after both Catholic and non-Catholic acquisitions. The one statistically significant result was employee benefit expenses per bed, which decreased by $4,890.66 (95% CI −8,235.41, −1,545.91) at non-Catholic-acquired hospitals compared to never-acquired hospitals. Supplemental Appendix Figure C5 shows the dynamic changes in employee benefit expenses per bed over time. There were no significant differences in treatment effect magnitudes after Catholic vs. non-Catholic acquisitions for any operating expense measure.

Similar to operating expenses, changes in employment were similar after both Catholic and non-Catholic acquisitions. Total FTEs per bed decreased after both Catholic and non-Catholic acquisitions relative to never-acquired hospitals, although this change was only significant at non-Catholic-acquired hospitals (ATT −0.26, 95% CI −0.43, −0.08). MD FTEs per bed decreased after both Catholic (−0.03, 95% CI −0.06, −0.01) and non-Catholic acquisitions (−0.02, 95% CI −0.03, −0.006) relative to never-acquired hospitals. Support staff FTEs per bed also decreased after acquisition, although this change was again only significant at non-Catholic-acquired hospitals compared to never-acquired hospitals (ATT −0.24, 95% CI −0.37, −0.11). The largest changes in employment post-acquisition were cuts to support staff, suggesting a large portion of the change in total FTEs were driven by a decrease in support staff. Differences in the changes after Catholic vs. non-Catholic acquisitions were not significant for any employment measure. The event study plots in Supplemental Appendix Figures C6–C8 are consistent with these ATT estimates and highlight the similarity in hospital employment changes after both Catholic and non-Catholic acquisitions.

### Robustness Checks

In Supplemental Appendix Tables D1–D4, we present the average treatment effects of Catholic and non-Catholic acquisitions for the following four robustness checks: two-way fixed effects estimator instead of imputation-based approach (Supplemental Appendix Table D1), 6 years pre- and post-period instead of 8 years (Supplemental Appendix Table D2), excluding Medicaid payer mix as a covariate (Supplemental Appendix Table D3), and adding entity type (government, nonprofit, and for-profit) as a covariate (Supplemental Appendix Table D4). Overall, results from these robustness checks were similar to our main DID models, although there were some divergences. When using the two-way fixed effects estimator, changes in chaplaincy and charity care were no longer statistically significant at Catholic-acquired hospitals compared to never-acquired hospitals (Supplemental Appendix Table D1). However, the probability of community outreach decreased by 3.34 pp (95% CI −6.13, −0.56) at non-Catholic-acquired hospitals compared to never-acquired hospitals. The only change when using 6 years pre- and post-period instead of 8 years was the difference in treatment effect magnitudes between Catholic-acquired vs. non-Catholic-acquired hospitals was no longer statistically significant for charity care (Supplemental Appendix Table D2, col. 3). The only change when removing Medicaid payer mix as a covariate was support staff FTEs per bed decreased by 0.41 (95% CI −0.80, −0.01) at Catholic-acquired hospitals compared to never-acquired hospitals (Supplemental Appendix Table D3). When adding entity type as a covariate, changes in employee benefit expenses per bed after non-Catholic acquisitions and changes in MD FTEs per bed after both Catholic and non-Catholic acquisitions were no longer statistically significant (Supplemental Appendix Table D4).

## Discussion

In recent decades, health system acquisitions have become a defining feature of the U.S. health care landscape, often driven by financial pressures and the need for operational efficiency. While prior research has highlighted the cost-cutting strategies commonly associated with acquisitions, less is known about the role of Catholic-owned systems—which constitute 4 of the 10 largest U.S. health systems—in shaping post-acquisition outcomes (Andreyeva et al., 2024; Solomon et al., 2020). This study leverages a national sample of U.S. hospitals over a 14-year period to provide new insights into the operational impacts of Catholic and non-Catholic health systems’ acquisitions. In doing so, we provide rigorous empirical evidence to inform ongoing state policy debates related to the expansion of Catholic health care and also make novel theoretical and methodological contributions.

We found that hospitals were more likely to provide some mission-oriented services, including chaplaincy and charity care, after being acquired by a Catholic vs. non-Catholic system (relative to the control group of never-acquired hospitals). However, there was no change in the likelihood of offering community outreach or linguistic/translation services after being acquired by a Catholic or non-Catholic system. In addition, hospitals acquired by non-Catholic systems were less likely to operate an obstetric unit and had lower obstetric bed count relative to never-acquired hospitals, while hospitals acquired by Catholic systems had no significant change in obstetric services after acquisition. These results generally support Hypothesis 1 and align with previous literature finding Catholic ownership was associated with providing more mission-oriented services (Weisman et al., 1999; White et al., 2006, 2010; Zare & Gabow, 2023).

Our results provide mixed support for Hypothesis 2. Aligning with expectations, changes in operating expenses and employment were generally similar after both Catholic and non-Catholic acquisitions. These results align with previous research showing reductions in operating costs, primarily by reducing employees in support functions, after previously independent hospitals join a health system (Andreyeva et al., 2024). In addition, these findings align with previous literature highlighting growing similarities between Catholic and non-Catholic hospitals in managerial/operating strategies (Homan & White, 2021) and quality measures (Kutney-Lee et al., 2014). However, contrary to Hypothesis 2 expectations, we found differential changes in some utilization outcomes (e.g., admissions per bed) after Catholic vs. non-Catholic acquisitions. Future research should investigate the organizational behavior driving these heterogeneous utilization results.

We take multiple steps to assess the validity of our estimates. First, we formally test and are unable to reject the null hypothesis of no differences in pretrends at treated (acquired) hospitals compared to the control group (never-acquired hospitals), implying the former were not on track to experience the observed changes absent treatment. Second, we confirm the robustness of our results to multiple study design and specification checks, increasing our confidence in the causal interpretation of findings.

### Health Policy and Practice Implications

Our findings provide practical evidence on the effects of health system ownership for policymakers and health care leaders. Health care consolidation faces increasing public scrutiny given the growing role of for-profit entities and industry incentives for profit-maximization, often prompting calls for enhanced government oversight (Furukawa et al., 2020; Fuse Brown & Gudiksen, n.d.). Catholic health system acquisitions present an interesting dynamic, as our findings show they have the potential to preserve or even expand some mission-oriented services. However, this also raises key questions about equitable access to services prohibited by Catholic doctrine, such as abortion and contraception. In the wake of the *Dobbs v. Jackson Women’s Health Organization* Supreme Court decision, access to comprehensive reproductive health care is particularly important. Future research should explore the impact of Catholic ownership on access to comprehensive reproductive health care, especially in areas where Catholic hospitals have significant market share.

In addition, our study makes novel theoretical and methodological contributions. Using the case of Catholic system acquisitions, we demonstrate how conflicting institutional logics interact and evolve over time within newly acquired hospitals. In some cases, mission-oriented logics outweigh financial logics—for example, expanding charity care at acquired hospitals. Aligned with institutional theories and organizational identity, the explicit nature of the ERDs and human/financial resources dedicated to their consistent implementation may play a role in the preservation of mission-oriented services (Besharov & Smith, 2014; David et al., 2019; White et al., 2006). Furthermore, this study utilizes an advanced imputation-based DID estimator, which is particularly well-suited for settings with staggered treatment timing and heterogeneous treatment effects. By employing this method, we provide robust causal inference and greater precision in estimating group-specific effects (e.g., Catholic vs. non-Catholic acquisitions). This approach advances the econometric literature by addressing limitations of traditional two-way fixed effects models and demonstrates the importance of evaluating ownership-based heterogeneity in system acquisitions.

### Limitations

This study has several limitations. First, although Catholic systems are the largest provider of religiously-affiliated care in the United States, they still only operate a minority of total hospitals. The relatively small number of Catholic acquisitions compared to non-Catholic acquisitions (76 vs. 631 hospitals) may reduce statistical power, leading to wider confidence intervals and potentially limiting the detection of significant effects. Second, this study examined hospital-level operations and does not utilize clinical data to verify the actual use or quality of services provided, which limits our ability to assess patient-level impacts. Third, our analytic sample compares first-time acquisitions with never-acquired hospitals to ensure a clean comparison and valid identification of post-acquisition effects. While this design enhances internal validity, it narrows generalizability by excluding hospitals with prior health system affiliations. Fourth, this analysis focuses on quantitative outcomes, leaving qualitative aspects of hospital operations, such as leadership decisions or cultural integration, unexplored. Finally, unobserved factors such as market conditions or leadership changes may confound some results despite the inclusion of covariates and fixed effects.

## Conclusion

This study uses rigorous quasi-experimental methods, informed by organizational theory, to examine how health care providers balance conflicting institutional pressures. We find that, while acquisition effects broadly align with the cost-containment strategies observed in prior research, there were heterogeneous effects based on system ownership. Some mission-oriented services, such as chaplaincy and charity care, were more likely to be provided after Catholic vs. non-Catholic acquisitions. In addition, non-Catholic systems were more likely to cut obstetric services at newly acquired hospitals. Taken together, these results suggest that system ownership plays a role in shaping hospital priorities post-acquisition and highlights the importance of disaggregating effects by ownership type. As health system consolidation continues, policymakers and health care leaders should consider the implications of these differing approaches, particularly in balancing financial sustainability with equitable access to care.

## Supplemental Material

sj-pdf-1-mcr-10.1177_10775587251355541 – Supplemental material for Mission vs. Margin: The Effects of Catholic Health System Ownership on Hospital Operations Supplemental material, sj-pdf-1-mcr-10.1177_10775587251355541 for Mission vs. Margin: The Effects of Catholic Health System Ownership on Hospital Operations by Alex Schulte, Becky Staiger, Hector P. Rodriguez and Amanda L. Brewster in Medical Care Research and Review

sj-pdf-2-mcr-10.1177_10775587251355541 – Supplemental material for Mission vs. Margin: The Effects of Catholic Health System Ownership on Hospital Operations Supplemental material, sj-pdf-2-mcr-10.1177_10775587251355541 for Mission vs. Margin: The Effects of Catholic Health System Ownership on Hospital Operations by Alex Schulte, Becky Staiger, Hector P. Rodriguez and Amanda L. Brewster in Medical Care Research and Review

sj-pdf-3-mcr-10.1177_10775587251355541 – Supplemental material for Mission vs. Margin: The Effects of Catholic Health System Ownership on Hospital Operations Supplemental material, sj-pdf-3-mcr-10.1177_10775587251355541 for Mission vs. Margin: The Effects of Catholic Health System Ownership on Hospital Operations by Alex Schulte, Becky Staiger, Hector P. Rodriguez and Amanda L. Brewster in Medical Care Research and Review

sj-pdf-4-mcr-10.1177_10775587251355541 – Supplemental material for Mission vs. Margin: The Effects of Catholic Health System Ownership on Hospital Operations Supplemental material, sj-pdf-4-mcr-10.1177_10775587251355541 for Mission vs. Margin: The Effects of Catholic Health System Ownership on Hospital Operations by Alex Schulte, Becky Staiger, Hector P. Rodriguez and Amanda L. Brewster in Medical Care Research and Review
