# Supplementary material for: Mission vs. Margin: The Effects of Catholic Health System Ownership on Hospital Operations
Source: Med Care Res Rev. 2025 Jul 24;82(6):465–76. doi: 10.1177/10775587251355541 (PMC12541111; doi:10.1177/10775587251355541)
Supplement: sj-pdf-1-mcr-10.1177_10775587251355541 – Supplemental material for Mission vs. Margin: The Effects of Catholic Health System Ownership on Hospital Operations [file sj-pdf-1-mcr-10.1177_10775587251355541.pdf]

APPENDIX D

Appendix D presents tables for the following robustness checks: standard two-way fixed effects difference-in-differences (DID) estimator rather than Borusyak et al. DID imputation-based approach (Table D1), limiting the pre- and post-periods to 6 years instead of 8 (Table D2), excluding Medicaid payer mix as a covariate (Table D3), and including hospital entity type (government, nonprofit, for-profit) as a covariate (Table D4). These Tables present the average treatment effects of Catholic (col. 1) and non-Catholic (col. 2) acquisitions. If present, column (3) uses an imputation approach to estimate the difference between Catholic-acquired (col. 1) and non-Catholic-acquired (col. 2) treatment effects.

Table D1: Treatment Effects After Catholic and Non-Catholic Acquisitions Using Two-Way Fixed Effects DID Estimator

	(1)	(2)
	**Catholic-acquired**	**Non-Catholic-acquired**
**MISSION-ORIENTED SERVICES**		
**Chaplaincy**	0.07	-0.004
	(0.04)	(0.01)
	[-0.01, 0.16]	[-0.03, 0.02]
**Charity care**	0.04	0.001
	(0.02)	(0.01)
	[-0.01, 0.09]	[-0.02, 0.02]
**Community outreach**	-0.04	-0.03*
	(0.04)	(0.01)
	[-0.12, 0.04]	[-0.06, -0.01]
**Linguistic/translation services**	-0.03	0.0003
	(0.04)	(0.01)
	[-0.11, 0.06]	[-0.03, 0.03]
**OBSTETRICS**		
**Obstetrics unit**	-0.06	-0.04***
	(0.03)	(0.01)
	[-0.12, 0.01]	[-0.07, -0.02]
**Obstetric bed count**	-0.67	-0.78***
	(0.38)	(0.22)
	[-1.42, 0.09]	[-1.2, -0.35]
**Proportion obstetric beds/total beds**	-0.005	-0.002
	(0.004)	(0.002)
	[-0.01, 0.004]	[-0.01, 0.001]
**UTILIZATION**		
**Bed count**	-11.23***	-3.26
	(3.16)	(1.81)
	[-17.42, -5.04]	[-6.82, 0.30]
**Admissions per bed**	-1.67	0.90*

	(1.05)	(0.40)
	[-3.73, 0.39]	[0.11, 1.68]
**Medicaid days per bed**	3.59	3.55**
	(2.94)	(1.03)
	[-2.17, 9.35]	[1.53, 5.57]
**Medicare days per bed**	-6.46*	2.62*
	(2.80)	(1.26)
	[-11.95, -0.96]	[0.16, 5.09]
**OPERATING EXPENSES**		
**Total expenses per bed**	15182.28	25948.78
	(43006.87)	(16147.88)
	[-69147.89, 99512.45]	[-5714.85, 57612.41]
**Payroll expenses per bed**	-6679.25	-4053.60
	(16926.51)	(6446.81)
	[-39869.65, 26511.17]	[-16694.85, 8587.64]
**Employee benefit expenses per bed**	-3406.00	-4380.84*
	(5057.19)	(1752.43)
	[-13322.40, 6510.41]	[-7817.09, -944.59]
**EMPLOYMENT**		
**Total FTEs per bed**	-0.36	-0.24**
	(0.30)	(0.09)
	[-0.94, 0.22]	[-0.40, -0.07]
**MD FTEs per bed**	-0.03	-0.01
	(0.02)	(0.01)
	[-0.07, 0.003]	[-0.02, 0.002]
**Nurse FTEs per bed**	0.002	0.01
	(0.07)	(0.02)
	[-0.14, 0.15]	[-0.04, 0.05]
**Support staff FTEs per bed**	-0.33	-0.22**
	(0.22)	(0.06)
	[-0.75, 0.1]	[-0.34, -0.09]

Notes: The model includes hospital and year fixed effects and controls for Medicaid payer mix. MD, Doctor of Medicine (includes physicians and dentists); FTE, full time equivalents. Standard errors are clustered at the hospital level and presented in parentheses. 95% confidence intervals are presented in brackets. * < 0.05 ** <0.01 *** <0.001

Table D2: Treatment Effects After Catholic and Non-Catholic Acquisitions with 6 Years Pre- and Post-Period

	(1)	(2)	(3)
	**Catholic-acquired**	**Non-Catholic-acquired**	**Difference between Catholic-acquired and non-Catholic-acquired**
**MISSION-ORIENTED SERVICES**			
**Chaplaincy**	0.09*	-0.001	0.09*
	(0.04)	(0.01)	(0.04)
	[0.005, 0.17]	[-0.03, 0.02]	[0.004, 0.17]
**Charity care**	0.04*	-0.0008	0.04
	(0.02)	(0.01)	(0.02)
	[0.001, 0.07]	[-0.02, 0.02]	[-0.002, 0.08]
**Community outreach**	-0.02	-0.03	0.004
	(0.04)	(0.01)	(0.04)
	[-0.10, 0.05]	[-0.05, 0.0006]	[-0.08, 0.08]
**Linguistic/translation services**	0.009	0.01	0.002
	(0.04)	(0.01)	(0.04)
	[-0.07, 0.09]	[-0.02, 0.04]	[-0.08, 0.08]
**OBSTETRICS**			
**Obstetrics unit**	-0.04	-0.04**	-0.002
	(0.03)	(0.01)	(0.03)
	[-0.09, 0.01]	[-0.06, -0.01]	[-0.06, 0.05]
**Obstetric bed count**	-0.63	-0.79***	0.15
	(0.36)	(0.21)	(0.41)
	[-1.34, 0.07]	[-1.20, -0.37]	[-0.65, 0.95]
**Proportion obstetric beds/total beds**	-0.004	-0.001	-0.002
	(0.004)	(0.002)	(0.004)
	[-0.01, 0.004]	[-0.005, 0.002]	[-0.01, 0.006]
**UTILIZATION**			
**Bed count**	-11.82***	-3.73*	-8.09*
	(2.83)	(1.76)	(3.21)
	[-17.37, -6.27]	[-7.18, -0.28]	[-14.39, -1.79]
**Admissions per bed**	-1.07	1.12**	-2.19*
	(0.94)	(0.42)	(1.01)
	[-2.90, 0.77]	[0.31, 1.94]	[-4.17, -0.22]
**Medicaid days per bed**	4.37	3.99***	0.38
	(2.32)	(1.08)	(2.48)
	[-0.18, 8.93]	[1.89, 6.10]	[-4.47, 5.23]
**Medicare days per bed**	-5.58*	3.07*	-8.64**
	(2.67)	(1.27)	(2.89)

	[-10.82, 0.34]	[0.57, 5.57]	[-14.30, -2.99]
**OPERATING EXPENSES**			
**Total expenses per bed**	27085.01	21321.50	5763.51
	(36205.39)	(14937.58)	(37828.21)
	[-43876.24, 98046.26]	[-7955.623, 50598.62]	[-68378.41, 79905.44]
**Payroll expenses per bed**	-4491.67	-5440.38	948.71
	(14341.55)	(5764.83)	(14936.87)
	[-32600.60, 23617.26]	[-16739.23, 5858.48]	[-28327.01, 30224.43]
**Employee benefit expenses per bed**	-2038.73	-4637.56**	2598.83
	(4061.50)	(1567.05)	(4208.44)
	[-9999.12, 5921.67]	[-7708.91, -1566.21]	[-5649.56, 10847.23]
**EMPLOYMENT**			
**Total FTEs per bed**	-0.31	-0.21*	-0.10
	(0.24)	(0.08)	(0.25)
	[-0.78, 0.16]	[-0.37, -0.05]	[-0.59, 0.38]
**MD FTEs per bed**	-0.03*	-0.01*	-0.02
	(0.01)	(0.005)	(0.01)
	[-0.06, -0.006]	[-0.02, -0.003]	[-0.04, 0.009]
**Nurse FTEs per bed**	0.006	0.02	-0.01
	(0.06)	(0.02)	(0.06)
	[-0.11, 0.12]	[-0.02, 0.06]	[-0.14, 0.11]
**Support staff FTEs per bed**	-0.28	-0.19**	-0.09
	(0.18)	(0.06)	(0.19)
	[-0.63, 0.07]	[-0.31, -0.08]	[-0.45, 0.27]

Notes: The model includes hospital and year fixed effects and controls for Medicaid payer mix. MD, Doctor of Medicine (includes physicians and dentists); FTE, full time equivalents. Standard errors are clustered at the hospital level and presented in parentheses. 95% confidence intervals are presented in brackets. * < 0.05 ** <0.01 *** <0.001

Table D3: Treatment Effects After Catholic and Non-Catholic Acquisitions without Medicaid Payer Mix Covariate

	(1)	(2)	(3)
	**Catholic-acquired**	**Non-Catholic-acquired**	**Difference between Catholic-acquired and non-Catholic-acquired**
**MISSION-ORIENTED SERVICES**			
**Chaplaincy**	0.10*	0.001	0.10*
	(0.05)	(.01)	(0.05)
	[0.01, 0.19]	[-0.03, 0.03]	[0.01, 0.20]
**Charity care**	0.04*	-0.001	0.04*
	(0.02)	(0.01)	(0.02)
	[0.005, 0.08]	[-0.02, 0.02]	[0.002, 0.08]
**Community outreach**	-0.003	-0.02	0.02
	(0.04)	(0.02)	(0.04)
	[-0.09, 0.08]	[-0.05, 0.006]	[-0.07, 0.11]
**Linguistic/translation services**	0.03	0.01	0.01
	(0.04)	(0.02)	(0.04)
	[-0.06, 0.11]	[-0.02, 0.04]	[-0.07, 0.10]
**OBSTETRICS**			
**Obstetrics unit**	-0.04	-0.03**	-0.01
	(0.03)	(0.01)	(0.03)
	[-0.09, 0.01]	[-0.06, -0.01]	[-0.06, 0.05]
**Obstetric bed count**	-0.78	-0.86***	0.07
	(0.42)	(0.22)	(0.46)
	[-1.61, 0.05]	[-1.29, -0.43]	[-0.83, 0.99]
**Proportion obstetric beds/total beds**	-0.005	-0.001	-0.004
	(0.004)	(0.002)	(0.005)
	[-0.01, 0.004]	[-0.005, 0.002]	[-0.01, 0.006]
**UTILIZATION**			
**Bed count**	-11.76***	-3.89*	-7.87*
	(3.11)	(1.86)	(3.49)
	[-17.86, -5.67]	[-7,53, -0.25]	[-14.72, -1.02]
**Admissions per bed**	-1.50	1.29**	-2.79*
	(1.03)	(0.44)	(1.10)
	[-3.51, 0.52]	[0.42, 2.16]	[-4.94, -0.63]
**OPERATING EXPENSES**			
**Total expenses per bed**	23746.76	21137.85	2608.91
	(39327.44)	(16544.45)	(41101.35)

	-53333.6, 100827.1]	[-11288.68, 53564.39]	[-77948.25, 83166.07]
**Payroll expenses per bed**	-8661.56	-6990.46	-1671.10
	(15288.95)	(6314.77)	(15912.70)
	[-38627.36, 21304.24]	[-19367.18, 5386.27]	[-32859.42, 29517.22]
**Employee benefit expenses per bed**	-4091.64	-5280.44**	1188.80
	(4297.24)	(1710.51)	(4452.54)
	[-12514.07, 4330.79]	[-8632.98, -1927.90]	[-7538.02, 9915.63]
**EMPLOYMENT**			
**Total FTEs per bed**	-0.46	-0.30**	-0.16
	(0.27)	(0.09)	(0.28)
	[-0.98, 0.06]	[-0.48, -0.12]	[-0.70, 0.38]
**MD FTEs per bed**	-0.04**	-0.02***	-0.02
	(0.01)	(0.006)	(0.01)
	[-0.06, -0.01]	[-0.03, -0.007]	[-0.04, 0.008]
**Nurse FTEs per bed**	-0.007	0.006	-0.01
	(0.07)	(0.02)	(0.07)
	[-0.14, 0.13]	[-0.04, 0.05]	[-0.15, 0.012]
**Support staff FTEs per bed**	-0.41*	-0.27***	-0.14
	(0.20)	(0.07)	(0.21)
	[-0.80, -0.01]	[-0.40, -0.14]	[-0.54, 0.27]

Notes: The model includes hospital and year fixed effects. MD, Doctor of Medicine (includes physicians and dentists); FTE, full time equivalents. Standard errors are clustered at the hospital level and presented in parentheses. 95% confidence intervals are presented in brackets. * < 0.05 ** <0.01 *** <0.001

Table D4: Treatment Effects After Catholic and Non-Catholic Acquisitions with Entity Type Covariate

	(1)	(2)	(3)
	**Catholic-acquired**	**Non-Catholic-acquired**	**Difference between Catholic-acquired and non-Catholic-acquired**
**MISSION-ORIENTED SERVICES**			
**Chaplaincy**	0.10*	0.004	0.10*
	(0.05)	(.01)	(0.05)
	[0.01, 0.19]	[-0.02, 0.03]	[0.005, 0.19]
**Charity care**	0.04*	-0.002	0.04*
	(0.02)	(0.01)	(0.02)
	[0.003, 0.08]	[-0.02, 0.02]	[0.0007, 0.08]
**Community outreach**	0.001	-0.02	0.02
	(0.04)	(0.01)	(0.04)
	[-0.08, 0.09]	[-0.05, 0.01]	[-0.07, 0.11]
**Linguistic/translation services**	0.03	0.02	0.02
	(0.04)	(0.02)	(0.05)
	[-0.05, 0.12]	[-0.02, 0.05]	[-0.07, 0.11]
**OBSTETRICS**			
**Obstetrics unit**	-0.04	-0.03**	-0.01
	(0.03)	(0.01)	(0.03)
	[-0.10, 0.006]	[-0.06, -0.01]	[-0.07, 0.04]
**Obstetric bed count**	-0.80	-0.86***	0.06
	(0.42)	(0.22)	(0.47)
	[-1.63, 0.04]	[-1.30, -0.43]	[-0.86, 0.98]
**Proportion obstetric beds/total beds**	-0.005	-0.0008	-0.004
	(0.004)	(0.002)	(0.005)
	[-0.01, 0.004]	[-0.004, 0.003]	[-0.01, 0.005]
**UTILIZATION**			
**Bed count**	-12.37***	-4.19*	-8.19*
	(3.11)	(1.86)	(3.48)
	[-18.46, -6.29]	[-7,83, -0.55]	[-15.00, -1.37]
**Admissions per bed**	-1.45	1.27**	-2.72*
	(1.04)	(0.45)	(1.11)
	[-3.50, 0.60]	[0.39, 2.14]	[-4.90, -0.53]
**Medicaid days per bed**	3.19	3.96**	-0.77
	(2.62)	(1.21)	(2.74)
	[-1.93, 8.32]	[1.59, 6.33]	[-6.15, 4.61]
**Medicare days per bed**	-7.96**	3.08*	-11.04**
	(2.99)	(1.35)	(3.17)

	[-13.81, -2.11]	[0.44, 5.71]	[-17.26, -4.82]
**OPERATING EXPENSES**			
**Total expenses per bed**	53364.05	32746.29	20617.76
	(40399.17)	(16891.40)	(41657.72)
	[-25816.87, 132545.00]	[-360.25, 65852.83]	[-61029.87, 102265.40]
**Payroll expenses per bed**	3328.88	-613.54	3942.41
	(15905.24)	(6429.51)	(16280.99)
	[-27844.82, 34502.57]	[-13215.13, 11988.06]	[-27967.73, 35852.56]
**Employee benefit expenses per bed**	-731.19	-3244.37	2513.19
	(4484.24)	(1753.64)	(4558.92)
	[-9520.13, 8057.75]	[-6681.45, 192.71]	[-6422.14, 11448.51]
**EMPLOYMENT**			
**Total FTEs per bed**	-0.27	-0.21*	-0.06
	(0.27)	(0.09)	(0.27)
	[-0.80, 0.26]	[-0.40, -0.03]	[-0.60, 0.48]
**MD FTEs per bed**	-0.02*	-0.01	-0.01
	(0.01)	(0.006)	(0.01)
	[-0.05, 0.007]	[-0.02, 0.001]	[-0.04, 0.02]
**Nurse FTEs per bed**	0.04	0.02	0.02
	(0.07)	(0.02)	(0.07)
	[-0.10, 0.17]	[-0.03, 0.07]	[-0.12, 0.15]
**Support staff FTEs per bed**	-0.27	-0.20**	-0.07
	(0.20)	(0.07)	(0.21)
	[-0.67, 0.12]	[-0.34, -0.07]	[-0.47, 0.34]

Notes: The model includes hospital and year fixed effects and controls for Medicaid payer mix and entity type (government, nonprofit, for-profit). MD, Doctor of Medicine (includes physicians and dentists); FTE, full time equivalents. Standard errors are clustered at the hospital level and presented in parentheses. 95% confidence intervals are presented in brackets. * < 0.05 ** <0.01 *** <0.001
