# Supplementary material for: Mission vs. Margin: The Effects of Catholic Health System Ownership on Hospital Operations
Source: Med Care Res Rev. 2025 Jul 24;82(6):465–76. doi: 10.1177/10775587251355541 (PMC12541111; doi:10.1177/10775587251355541)

APPENDIX C

Appendix C presents event study plots for eight of our secondary outcomes (Figures C1-C8). These plots illustrate the dynamic trends in the average treatment effect on the treated (ATT) for Catholic-acquired and non-Catholic-acquired hospitals in the 8 years pre- and post-acquisition.

Figure C1: Change in number of total beds

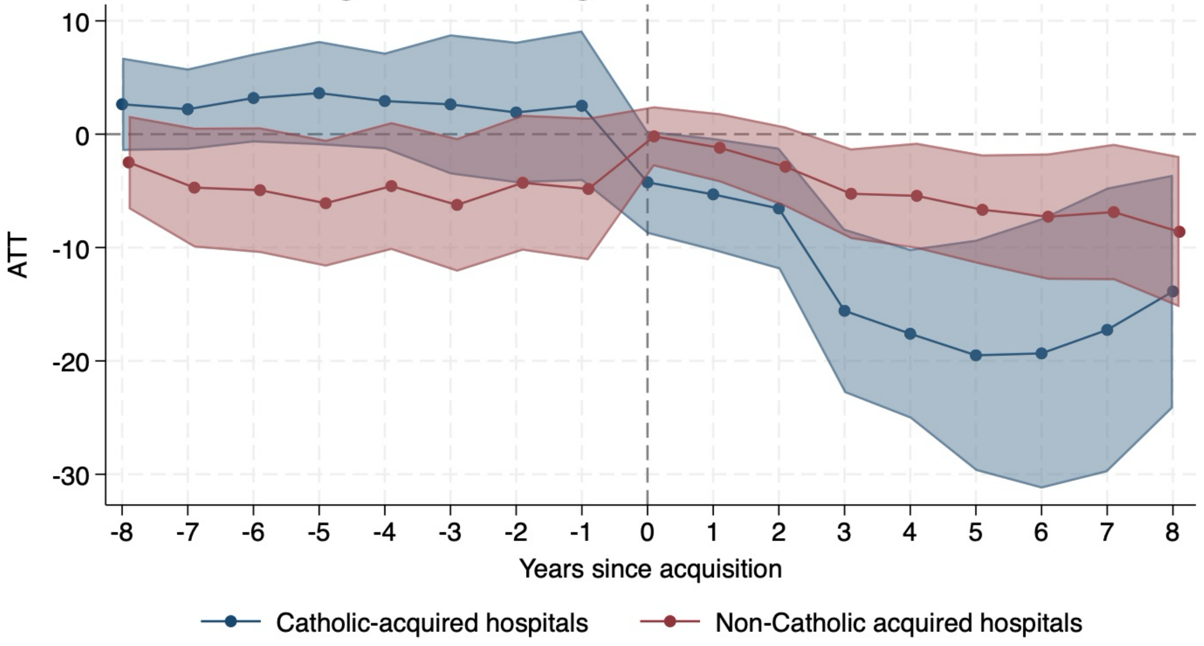

Figure C2: Change in admissions per bed

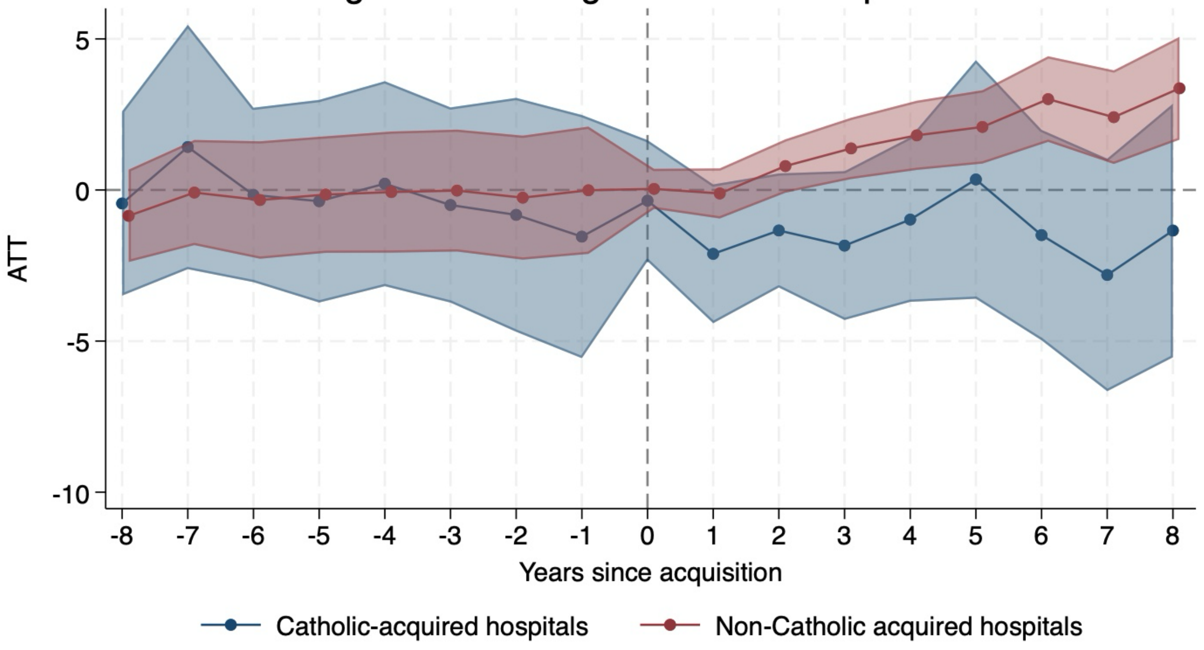

Figure C3: Change in Medicaid days per bed

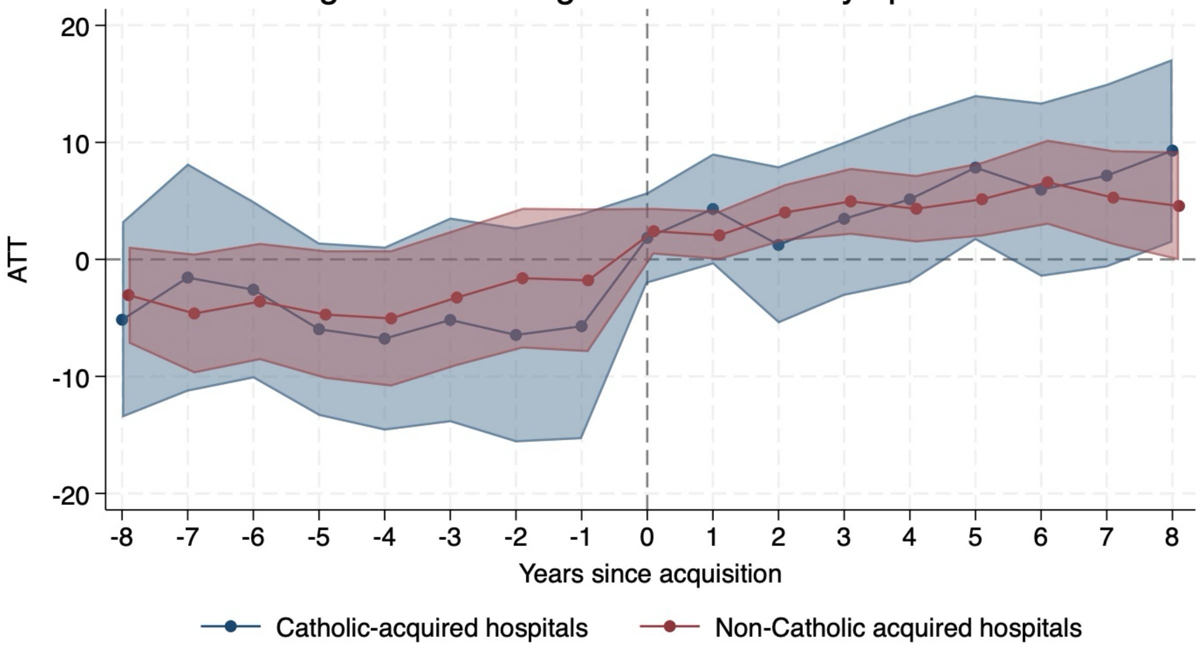

Figure C4: Change in Medicare days per bed

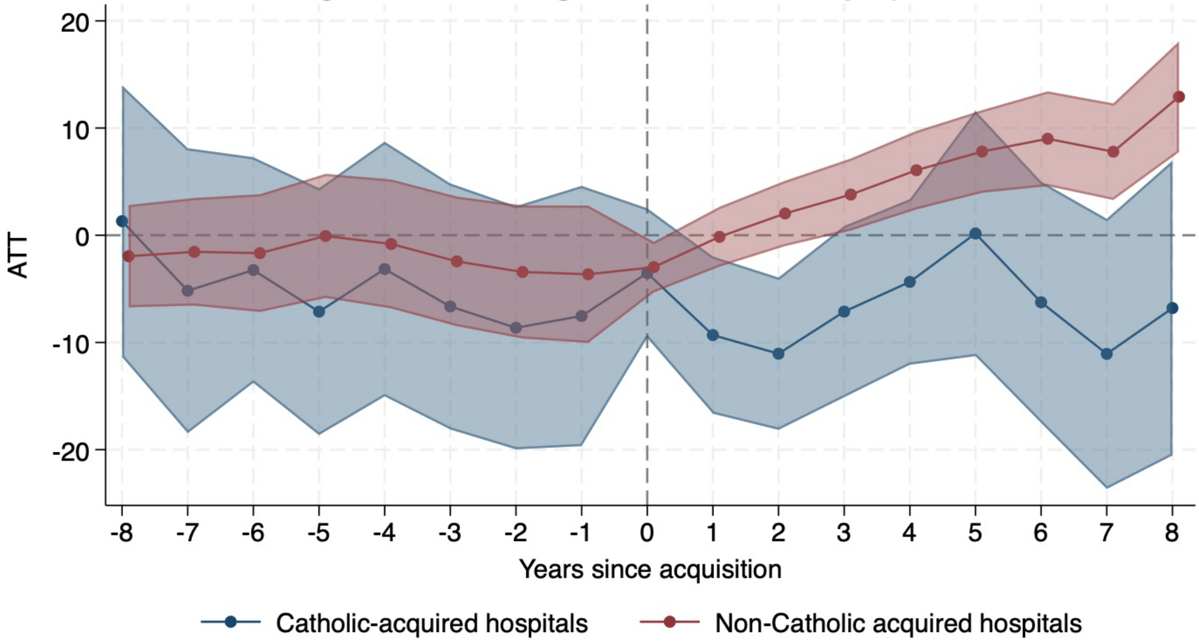

Figure C5: Change in employee benefit expenses per bed

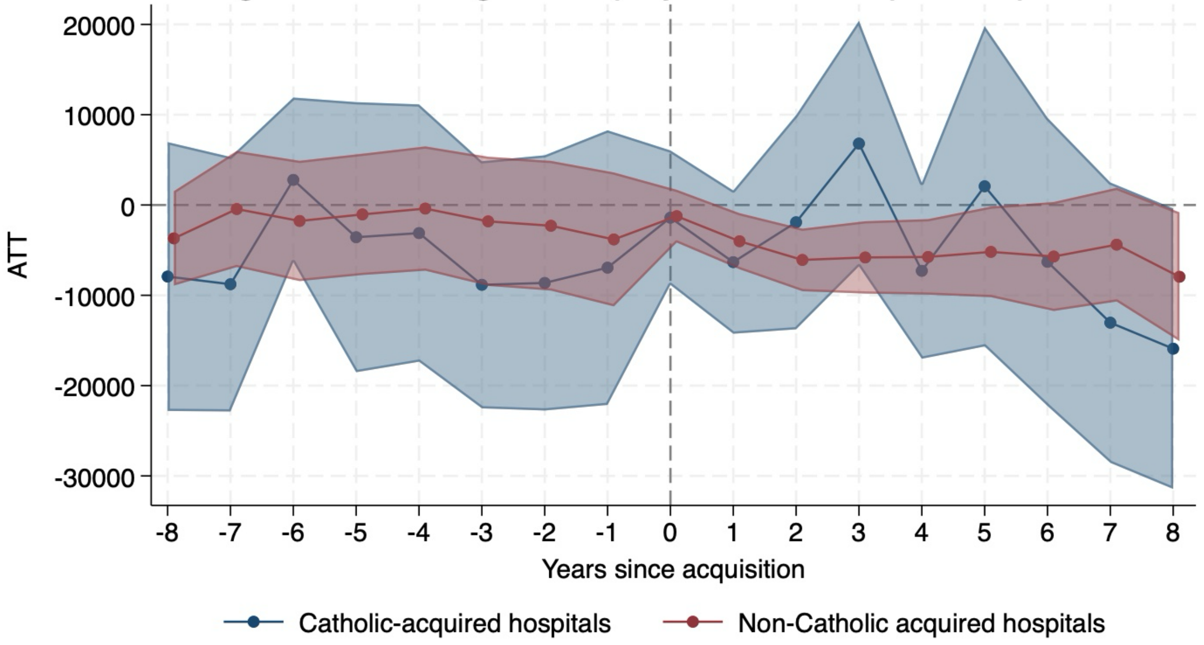

Figure C6: Change in total FTEs per bed

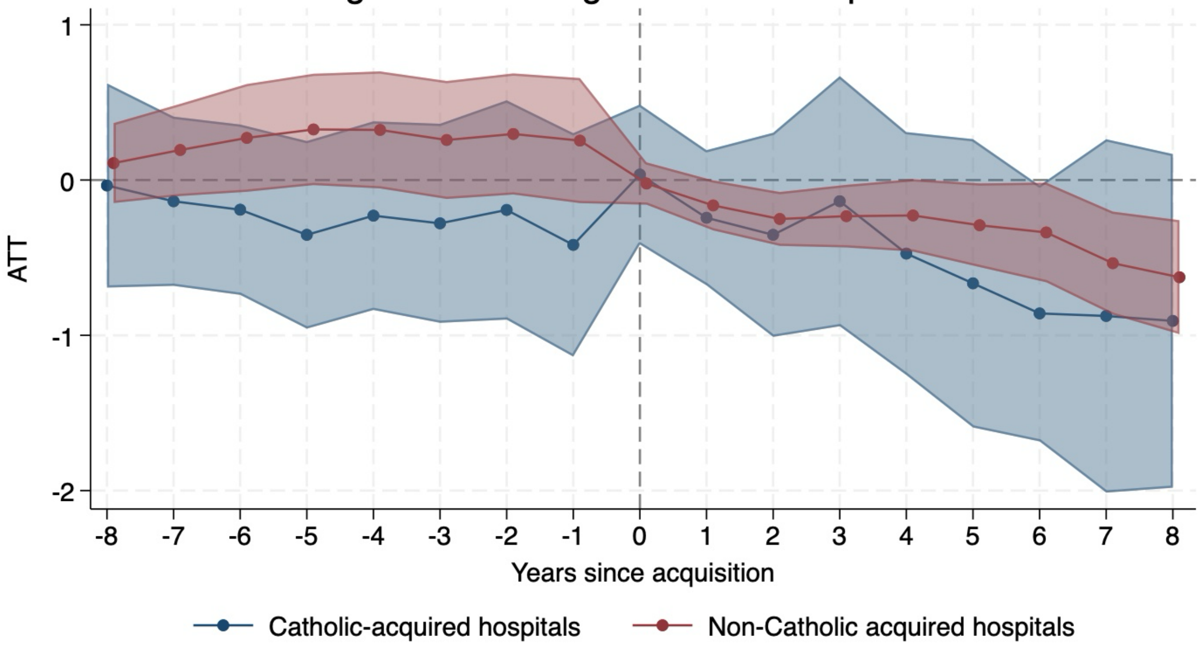

Figure C7: Change in MD FTEs per bed

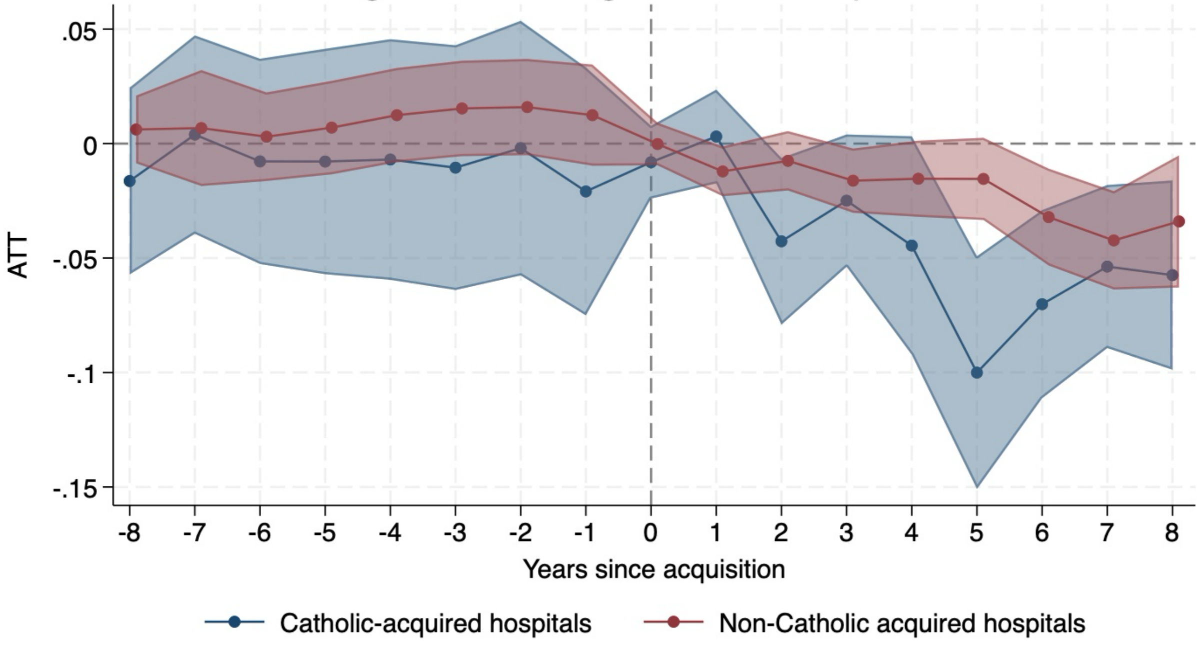

Figure C8: Change in support staff FTEs per bed

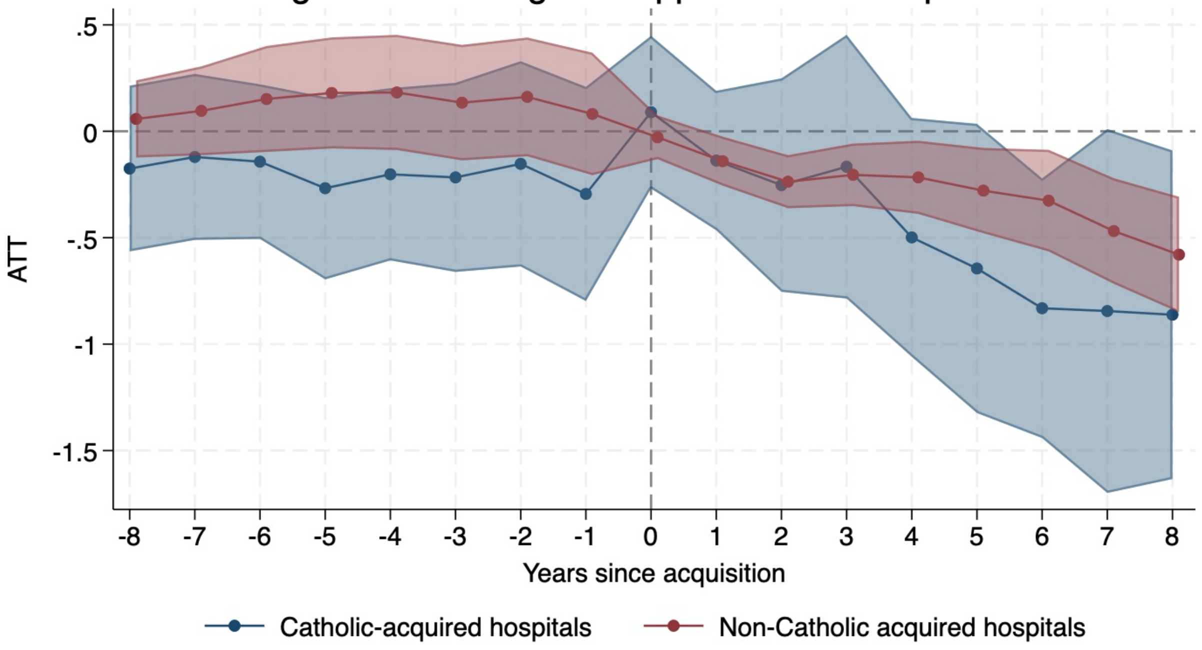

Supplement: sj-pdf-2-mcr-10.1177_10775587251355541 – Supplemental material for Mission vs. Margin: The Effects of Catholic Health System Ownership on Hospital Operations [file sj-pdf-2-mcr-10.1177_10775587251355541.pdf]
