# Supplementary material for: Mission vs. Margin: The Effects of Catholic Health System Ownership on Hospital Operations
Source: Med Care Res Rev. 2025 Jul 24;82(6):465–76. doi: 10.1177/10775587251355541 (PMC12541111; doi:10.1177/10775587251355541)
Supplement: sj-pdf-3-mcr-10.1177_10775587251355541 – Supplemental material for Mission vs. Margin: The Effects of Catholic Health System Ownership on Hospital Operations [file sj-pdf-3-mcr-10.1177_10775587251355541.pdf]

APPENDIX B

Appendix Table B1 presents *p*-values from formal statistical tests of jointly significant pre-trends for each outcome. We compare trends in the pre-period between the control group (“never-treated” independent hospitals) and various treatment groups: hospitals later acquired by any system type (col. 1), hospitals later acquired by Catholic systems (col. 2), and hospitals later acquired by non-Catholic systems (col. 3). Joint *p*-values of > 0.05 support the parallel pre-trends assumption, as we are unable to reject the null hypothesis of no differences in trends between treatment and control groups prior to acquisition. We control for Medicaid payer mix in our main difference-in-differences (DID) analyses given that trends were not parallel between the treatment and control groups as show in Table B1.

Table B1. Pre-trends joint *p*-values to test parallel trends assumption

	(1)	(2)	(3)
	**Any acquisition**	**Catholic-acquired**	**Non-Catholic-acquired**
**Mission-oriented services**			
Chaplaincy	0.85	0.40	0.69
Charity care	0.43	0.58	0.39
Community outreach	0.14	0.43	0.09
Linguistic/translation services	0.32	0.66	0.12
**Obstetrics**			
Obstetrics unit	0.74	0.08	0.93
Obstetric bed count	0.56	0.68	0.55
Proportion obstetric beds/total beds	0.92	0.27	0.86
**Utilization**			
Bed count	0.30	0.41	0.19
Admissions per bed	0.64	0.66	0.73
Medicaid days per bed	0.04*	0.09	0.05
Medicare days per bed	0.44	0.10	0.46
**Expenses**			
Total expenses per bed	0.68	0.29	0.83
Payroll expenses per bed	0.63	0.43	0.74
Employee benefit expenses per bed	0.27	0.15	0.32
**Employment**			
Total FTEs per bed	0.76	0.23	0.76
MD FTEs per bed	0.79	0.12	0.85
Nurse FTEs per bed	0.77	0.63	0.57
Support staff FTEs per bed	0.27	0.53	0.35
**Covariates**			
Medicaid payer mix	0.01*	0.65	0.002**

Notes: * < 0.05 ** <0.01 *** <0.001
