# Supplementary material for: Mission vs. Margin: The Effects of Catholic Health System Ownership on Hospital Operations
Source: Med Care Res Rev. 2025 Jul 24;82(6):465–76. doi: 10.1177/10775587251355541 (PMC12541111; doi:10.1177/10775587251355541)
Supplement: sj-pdf-4-mcr-10.1177_10775587251355541 – Supplemental material for Mission vs. Margin: The Effects of Catholic Health System Ownership on Hospital Operations [file sj-pdf-4-mcr-10.1177_10775587251355541.pdf]

APPENDIX A

Targeting Regression

Analytical strategy

To better understand if Catholic and non-Catholic systems targeted different types of hospitals for acquisition, we began by employing a logistic regression model to examine hospital characteristics associated with acquisition, as specified by the following equation:

$$Y_{ht} = \beta X_{ht} + \theta_s + \theta_t + \epsilon_{ht}$$

In this model, the sample is restricted to independent hospitals, therefore excluding years after treated hospitals were acquired. For to-be-acquired hospitals, Y_{ht} was set to 1 for hospital *h* in year *t* the year before acquisition (and zero otherwise) so that hospital characteristics just prior to acquisition were used in the model prediction. We ran separate models by system ownership type to understand if different hospital characteristics were associated with acquisition by Catholic and non-Catholic health systems. $\mathbf{X_{ht}}$ is a vector of hospital characteristics that conceptually could predict system acquisition, including bed count, Medicaid payer mix, admissions per bed, entity type (government, non-profit, for-profit), rurality (metro, micro, rural), and dichotomous indicators for operating an obstetric unit and chaplaincy program. θ_s are state fixed effects and θ_t are year fixed effects. Standard errors were clustered at the state-level.

Results

Catholic systems were more likely to target hospitals that provided chaplaincy care (aOR 1.80; 95% confidence interval, CI, 1.11, 2.94) and less likely to target hospitals with higher Medicaid payer mix (aOR 0.14; 95% CI 0.03, 0.67) and in a rural location compared to metro (aOR 0.47; 95% CI 0.23, 0.94). In comparison, non-Catholic systems were more likely to target hospitals with higher bed count (aOR 1.23; 95% CI 1.08, 1.41), higher admissions per bed (aOR 1.01; 95% CI 1.01, 1.02), and an obstetrics unit (aOR 1.37; 95% CI 1.01, 1.84). No variable was a predictor of both Catholic and non-Catholic acquisitions.

Table A1. Targeting regression estimating hospital characteristics associated with Catholic and non-Catholic health system acquisition

	(1)	(2)
	**Catholic acquisition**	**Non-Catholic acquisition**
**Bed count**	0.79	1.23**
	(0.12)	(0.08)
	[0.59, 1.06]	[1.08, 1.41]
**Medicaid payer mix**	0.14*	0.70
	(0.11)	(0.18)
	[0.03, 0.67]	[0.41, 1.17]
**Admissions per bed**	1.01	1.01***
	(0.01)	(0.003)

	[0.99, 1.03]	[1.01, 1.02]
**Obstetrics unit**	0.66	1.37*
	(0.24)	(0.21)
	[0.32, 1.36]	[1.01, 1.84]
**Chaplaincy**	1.80*	1.12
	(0.45)	(0.13)
	[1.11, 2.94]	[0.89, 1.41]
**Entity type**		
**Non-profit**	(ref.)	(ref.)
**Government**	0.74	0.42***
	(0.24)	(0.06)
	[0.40, 1.38]	[0.31, 0.57]
**For-profit**	0.32	1.40
	(0.23)	(0.27)
	[0.08, 1.30]	[0.95, 2.06]
**Urbanicity**		
**Metro**	(ref.)	(ref.)
**Micro**	0.60	1.17
	(0.22)	(0.16)
	[0.3, 1.22]	[0.89, 1.53]
**Rural**	0.47*	1.03
	(0.17)	(0.19)
	[0.23, 0.94]	[0.72, 1.48]

Notes: Table A1 presents the results of a targeting regression estimating the adjusted odds ratios of hospital characteristics associated with Catholic system acquisition (col. 1) and non-Catholic system acquisition (col. 2). The sample is restricted to independent hospitals, therefore excluding years after treated hospitals were acquired. Bed count has been log transformed which is more suitable for logistic regression models. Standard errors are clustered by state and presented in parentheses. 95% confidence intervals are presented in brackets. * < 0.05 ** <0.01 *** <0.001

Discussion

Aligning with expectations, Catholic systems were more likely to target hospitals with chaplaincy programs. However, in contrast to anecdotal evidence (Doderer, 2011; Minda, 2020; Santos, 2023), we find that Catholic systems were less likely to target hospitals in rural areas (compared to metro areas), and also less likely to target hospitals with a higher Medicaid payer mix. Consistent with previous literature on health system behavior (Andreyeva et al., 2024; Sullivan & Gustafson, 2023), non-Catholic systems were more likely to target hospitals with more beds and more admissions per bed. Interestingly, no hospital characteristic was predictive of both Catholic and non-Catholic system acquisition. Future research should investigate the mechanisms driving the different targeting behavior of Catholic and non-Catholic systems.

Baseline Hospital Summary Statistics

Table A2 provides baseline summary statistics using the first year we observe each hospital (2009 in most cases). We present descriptive statistics for the control group of never-acquired hospitals (col. 1) and treated groups of Catholic-acquired hospitals and non-Catholic-acquired hospitals (col. 2 and 3, respectively). By incorporating hospital fixed effects, our DID model relies on "within-hospital" comparisons, effectively controlling for any time-invariant hospital characteristics that drive these baseline differences and may be correlated with our outcomes of interest.

Appendix Table A2: Baseline hospital summary statistics

	(1)	(2)	(3)
	**Never-acquired hospitals**	**Catholic-acquired hospitals**	**Non-Catholic-acquired hospitals**
**Mission-oriented services**			
Chaplaincy (%, n)	32.56 (656)	47.37 (36)	59.11 (373)
Charity care (%, n)	8.54 (172)	3.95 (3)	15.06 (95)
Community outreach (%, n)	36.92 (744)	59.21 (45)	62.60 (395)
Linguistic/translation (%, n)	30.67 (618)	43.42 (33)	47.54 (300)
**Obstetrics**			
Obstetrics unit (%, n)	29.83 (601)	48.68 (37)	57.05 (360)
Obstetric bed count (mean, SD)	4.18 (10.18)	6.11 (9.76)	11.10 (14.8)
Proportion obstetric beds/total beds (mean, SD)	0.03 (0.06)	0.06 (0.07)	0.06 (0.07)
**Utilization**			
Bed count (mean, SD)	107.64 (143.17)	91.86 (105.41)	167.32 (162.71)
Admissions per bed (mean, SD)	28.42 (18.65)	35.15 (18.64)	38.53 (17.96)
Medicaid days per bed (mean, SD)	43.67 (52.57)	27.95 (35.52)	44.37 (42.90)
Medicare days per bed (mean, SD)	71.93 (51.21)	97.05 (47.34)	102.24 (45.54)
**Operating expenses**			
Total expenses per bed (mean, SD)	634608.20 (572458.00)	688466.70 (403139.60)	732703.90 (460918.70)
Payroll expenses per bed (mean, SD)	261479.10 (216284.20)	285606.90 (168163.60)	302725.50 (181605.10)
Employee benefit expenses per bed (mean, SD)	66063.95 (61904.66)	78231.72 (58734.69)	79833.19 (53930.00)
**Employment**			
Total FTEs per bed (mean, SD)	5.16 (3.54)	5.73 (2.56)	5.65 (2.85)
MD FTEs per bed (mean, SD)	0.10 (0.18)	0.08 (0.14)	0.11 (0.19)
Nurse FTEs per bed (mean, SD)	1.40 (0.96)	1.54 (0.64)	1.56 (0.75)
Support staff FTEs per bed (mean, SD)	3.69 (2.53)	4.10 (1.92)	4.00 (2.05)
**Observations**			
Number of hospitals	2,015	76	631

Notes: All hospitals were independent in first year observed. All summary statistics are at the hospital-year level.

References

Andreyeva, E., Gupta, A., Ishitani, C., Sylwestrzak, M., & Ukert, B. (2024). The Corporatization of Independent Hospitals. *Journal of Political Economy Microeconomics*, *2*(3), 602–663. https://doi.org/10.1086/730454

Doderer, M. (2011). Catholic Hospitals and the Safety Net. *AMA Journal of Ethics*, *13*(8), 569–570. https://doi.org/10.1001/virtualmentor.2011.13.8.mhst1-1108

Minda, J. (2020, March 1). HSHS preserves rural health care through hospital acquisitions [Catholic Health Association of the United States]. *Catholic Health World*. https://www.chausa.org/publications/catholic-health-world/archive/article/march-1-2020/hshs-preserves-rural-health-care-through-hospital-acquisitions

Santos, A. J. (2023). Acquisitions and Partnerships Between Secular and Catholic Health Organizations: Navigating the Canonical, Ecclesial and Theological Considerations. *Catholic Health Association of the United States*, *17*(2). https://www.chausa.org/docs/default-source/hceusa/acquisitions-and-partnerships-between-secular-and-catholic-health-organizations.pdf?sfvrsn=79dfddf2_6

Sullivan, M., & Gustafson, K. (2023, February 13). Characteristics of Hospitals Undergoing Mergers and Acquisitions. *Avalere*. https://avalere.com/insights/characteristics-of-hospitals-undergoing-mergers-and-acquisitions
